# Qiangjing tablets ameliorate asthenozoospermia via mitochondrial ubiquitination and mitophagy mediated by LKB1/AMPK/ULK1 signaling

**DOI:** 10.1080/13880209.2023.2168021

**Published:** 2023-01-19

**Authors:** Guangsen Li, Yuanjie Xu, Yingxi Li, Degui Chang, Peihai Zhang, Ziyang Ma, Di’ang Chen, Yaodong You, Xiaopeng Huang, Jian Cai

**Affiliations:** Department of Urology, Hospital of Chengdu University of Traditional Chinese Medicine, Chengdu, China

**Keywords:** Mitophagy, LKB1/AMPK/ULK1, sperm motility, mitochondria, traditional Chinese medicine

## Abstract

**Context:**

Therapeutic effects of Qiangjing tablets (QJT) on sperm vitality and asthenozoospermia (AZS) have been confirmed. However, the mechanism of action remains unclear.

**Objective:**

This study investigates the effects of QJT on AZS and the underlying mechanism of action.

**Materials and methods:**

Sixty Sprague–Dawley rats were randomly divided into six groups: Control, ORN (ornidazole; 200 mg/kg), ORN + QJT-low (0.17 g/mL), ORN + QJT-middle (0.33 g/mL), ORN + QJT-high (0.67 g/mL), and ORN + QJT + Radicicol (0.67 g/mL QJT and 20 mg/kg radicicol) groups. Pathological evaluation and analysis of mitophagy were conducted by H&E staining and transmission electron microscopy, respectively. Reactive oxygen species were detected by flow cytometry. Protein expression was determined by Western blotting.

**Results:**

QJT significantly improved ORN-treated sperm motility and kinematic parameters, as well as the pathological symptoms of testicular and epididymal tissues. In particular, QJT mitigated impaired mitochondrial morphology, and increased the PHB, Beclin-1, LC3-II protein, and ROS levels (*p* < 0.05), and reduced the protein expression levels of LC3-I and p62 (*p* < 0.05). Mechanistically, QJT antagonized the downregulation of SCF and Parkin protein levels (*p* < 0.05). Furthermore, QJT significantly increased the protein expressions levels of LKB1, AMPKα, p-AMPKα, ULK1 and p-ULK1 (*p* < 0.05). The ameliorative effect of QJT on pathological manifestations, mitochondrial morphology, and the expressions of mitophagy and mitochondrial ubiquitination-related proteins was counteracted by radicicol.

**Discussion and conclusions:**

QJT improved AZS *via* mitochondrial ubiquitination and mitophagy mediated by the LKB1/AMPK/ULK1 signaling pathway. Our study provides a theoretical basis for the treatment of AZS and male infertility.

## Introduction

Infertility is a common reproductive disorder with increasing morbidity that affects many couples worldwide (Moore and Reijo-Pera 2000; Vander Borght and Wyns 2018). Male infertility, which mainly manifests as abnormalities in sperm concentration, motility, and morphology, accounts for approximately half of all infertility cases (Hwang et al. 2011). Asthenozoospermia (AZS), characterized by low sperm motility (progressive and non-progressive sperm motility <40%, or progressive sperm motility <32%), has been demonstrated to contribute to 40% of male infertility (Liu et al. 2015). The occurrence of AZS involves numerous factors, such as chromosome malformation, perturbed gene modulation, endocrine imbalance, and factors related to infection, immunity, and the environment (Balkan et al. 2008). Microsurgery and auxiliary reproductive technology are the main therapeutic approaches for treating male infertility (Agostini et al. 2017; Punjani et al. 2021). Various types of drugs are used to treat male infertility, but their therapeutic effects remain suboptimal (Chehab et al. 2015). Thus, developing effective drugs to treat AZS and male infertility is necessary.

Qiangjing tablets (QJT) were developed by the Affiliated Hospital of Chengdu University of Traditional Chinese Medicine and contain the following component herbs: *Panax ginseng* C. A. Mey. (Araliaceae), *Cuscuta chinensis* Lam. (Convolvulaceae), *Lycium barbarum* L. (Solanaceae), *Epimedium brevicornu* Maxim. (Berberidaceae), *Angelica sinensis* (Oliv.) Diels (Umbelliferae), *Leonurus japonicus* Houtt. (Lamiaceae), *Curculigo orchioides* Gaertn. (Amaryllidaceae), and *Plantago asiatica* L. (Plantaginaceae). They have been used to treat male infertility in clinical practice for more than 20 years (Xiong et al. 2009; Zhang P et al. 2018). Many studies have reported that QJT could improve sperm motility, reduce sperm malformation rates, and restore testosterone levels in male infertility patients. Our laboratory confirmed that QJT enhanced sperm vitality in patients with kidney deficiency and blood stasis syndrome *via* the TGF-β1/Smads pathway (You et al. 2020). Our previous study revealed that QJT improved infertility in male rats through the Fas/FasL pathway, indicated by increased sperm concentration, vigor and viability, as well as decreased apoptosis and FasL levels in spermatogenic cells (Zhang PH et al. 2016). In addition, the Shenfu Qiangjing decoction, which includes the compositions of QJT, reduced the sperm liquefaction time and relieved the weak levels of testosterone resulting from various kidney-yang deficiency symptoms (Xiong et al. 2009). Moreover, the effect of QJT on AZS was recently revealed by our group. We discovered that QJT enhanced reproductive function by modulating oxidative stress and apoptosis *via* the Keap/Nrf2 pathway in AZS rats (Li G, Zhang, et al. 2021). Based on our study, Toll-like (Yu et al. 2021) and MAPK (Li GS et al. 2018) signaling pathways are also involved in the effect of QJT on the AZS. In addition, QJT prevented blood-testis barrier injury through the PI3K/Akt/Rictor signaling pathway (Shen et al. 2022). These findings suggest that QJT regulates multiple biological processes in AZS and other infertility-related diseases.

Furthermore, our results have shown that QJT alleviated the mitochondrial membrane potential (MMP), ROS, and morphology of mitochondria in rats with AZS (Li GS et al. 2018), implying that QJT may affect the biological processes associated with damaged mitochondria.

Mitophagy is a commonly observed mechanism for removing impaired mitochondria and is associated with MMP and oxidative stress (Narendra 2021). In normal mitochondria, PINK1 is a mitochondrial serine/threonine kinase that is constitutively input into the mitochondria and is rapidly cleaved and degraded (Killackey et al. 2020). After MMP is depolarized, full-length PINK1 accumulates. The accumulation of PINK1 with kinase activity is sufficient to promote Parkin recruitment to the mitochondrial surface. After Parkin is recruited, Parkin-mediated ubiquitination of mitochondrial substrates induces mitochondrial autophagy. Parkin enhances the recruitment of ubiquitin-binding adaptor p62. The p62 protein not only aggregates ubiquitinated proteins by polymerizing with other p62 molecules but also recruits ubiquitinated products into autophagosomes by binding to LC3. p62 accumulates in mitochondria, binds to Parkin’s ubiquitinated mitochondrial substrate, mediates mitochondrial aggregation, and connects the ubiquitinated substrate with LC3, promoting the autophagic degradation of ubiquitinated proteins (Youle and Narendra 2011). Mitophagy participates in the physiological and pathological progression of sperm (Song et al. 2016). Hence, we hypothesized that QJT may alleviate AZS *via* mitophagy.

Therefore, this study explored the mechanism involved in the mitophagy of QJT in mitigating AZS in male rats and demonstrated that QJT improved AZS through mitochondrial ubiquitination and mitophagy mediated by the LKB1/AMPK/ULK1 signaling pathway.

## Materials and methods

### Animals

Adult male Sprague-Dawley (SD) rats (200–220 g) were purchased from DOSSY (Chengdu Dossy Experimental Animals Co., Ltd., Sichuan, China) and adapted to standard laboratory conditions for 7 d before the experiments. Rats were fed a standard diet and water *ad libitum* with 40%-60% relative humidity and a 12 h light-dark cycle at 25 ± 2 °C. All procedures were performed in accordance with the Guide for the Care and Use of Laboratory Animals (National Research Council Committee for the Update of the Guide for the Care and Use of Laboratory 2011), and the ethical standards were approved by the ethical committee of the West China Hospital, Sichuan University (approval number: 20211304 A).

### Groups and treatments

Sixty SD rats were randomly divided into six groups: control, ORN, ORN + QJT-low, ORN + QJT-middle, ORN + QJT-high, and ORN + QJT + radicicol. The AZS model was constructed using ornidazole (ORN). Rats in the control and ORN groups were gavaged with 200 mg/kg/d ORN [dissolved in 1% sodium carboxymethylcellulose (CMC-Na, Solarbio, Beijing, China)] and the same amount of 1% CMC-Na for six consecutive weeks. Rats in ORN + QJT-low, ORN + QJT-middle and ORN + QJT-high were gavaged with 200 mg/kg/d ORN for three consecutive weeks and then gavaged with QJT [its preparation shown in our recent study (Li G, Zhang, et al. 2021)] at a concentration of 0.17, 0.33, and 0.67 g/mL before ORN administration in weeks 4–6 respectively. Rats in ORN + QJT + radicicol were gavaged with 200 mg/kg/d ORN for three consecutive weeks and then gavaged with 0.67 g/mL QJT and synchronously injected intraperitoneally with 20 mg/kg radicicol (CAS NO. 12772-57-5, AdooQ BioScience, CA, USA) in weeks 4–6. After the last treatment for 12 h, rats were intraperitoneally anesthetized with 25% ethyl carbamate (4 mL/kg) to obtain testis tissues, which were removed and stored for subsequent assays.

### Analysis of sperm parameters

The bilateral epididymal tissues were placed in pre-heated Hams’ F10 culture medium (Solarbio) and incubated for 30 min at 37 °C until the epididymal sperm were completely separated. Sperm motility was assessed using a Suiplus SSA-II automatic sperm detection and analysis system (Suiplus, Beijing, China). The kinematic parameters of sperm motility were determined using the TOX IVOS sperm analyzer system (Hamilton Thorne Biosciences, Beverly, MA, USA) after the sperm cells were collected from the vas deferens with a mild mixture (Cordero-Martínez et al. 2014).

### Histological analysis

Testis and epididymis tissues were removed, fixed in 4% formaldehyde, dehydrated, embedded, and sectioned. The sections were then stained with hematoxylin and eosin (H&E). The stained sections were imaged under a light microscope (Olympus, Tokyo, Japan), and images were evaluated using Image-Pro Plus 6.0 software (Media Cybernetics, USA).

### Transmission electron microscopy (TEM)

Testis and epididymis tissues were fixed in 3% glutaraldehyde and 1% osmium tetroxide and cut using an ultramicrotome. The sections were then continuously stained with 1% uranyl acetate and 0.5% lead citrate. Images were captured using a JEM-1400PLUS transmission electron microscope.

### ROS detection

ROS levels were analyzed using a Reactive Oxygen Species Assay kit (S0033, Beyotime, Shanghai, China) according to the manufacturer’s instructions. Fluorescence was determined using flow cytometry (BD FACSVerse).

### Western blotting

The total proteins in the testis tissues were separated using a Total Protein Extraction Kit (BC3711, Solarbio). The protein concentration was measured using a Protein Assay kit (Beyotime). Next, the protein samples were separated by 10% SDS-PAGE and electrically transferred to PVDF membranes (Millipore, MA, USA). After sealing with 3% bovine serum albumin at room temperature for 1 h, the membranes were hatched with the primary antibodies (rabbit anti-prohibitin (PHB), ab28172, 1:1000; rabbit anti-Beclin-1, ab62557, 1:2000; rabbit anti-LC3II/I, ab128025, 1:1,000; rabbit anti-p62, ab56416, 1:1,000; rabbit anti-SCF, ab64677, 1:1000; rabbit anti-Parkin, ab15494, 1:1,000; rabbit anti-LKB1, ab199970, 1:1,000; rabbit anti-AMPKα, ab131512, 1:500; rabbit anti-phosphorylated AMPKα (p-AMPKα), ab133448, 1:10000; rabbit anti-ULK1, ab167139, 1:1000; rabbit anti-p-ULK1, ab203207, 1:1000; rabbit anti-β-actin, ab8227, 1:5000; Abcam, Cambridge, UK) overnight at 4 °C. The membranes were incubated with goat-anti-rabbit IgG (H + L)-HRP (1:10000, ab6721, Abcam) for 1 h at room temperature and then rinsed thrice with TBST thrice. Protein bands were visualized using an Electrochemilluminescence (ECL) chemiluminescence kit (WBULS0500; EMD Millipore), and band intensity was analyzed with Image-Pro Plus 6.0.

### Statistical analysis

Data were shown as means ± standard deviation (SD). Differences were determined by one-way analysis of variance and Duncan’s test using the SPSS 20.0 package (SPSS Inc. Chicago, IL, USA). Differences were regarded as statistically non-significant and significant when *p* > 0.05 and *p* < 0.05, respectively.

## Results

### QJT played a positive role in sperm parameters of ORN-treated rats

**Figure 1.**
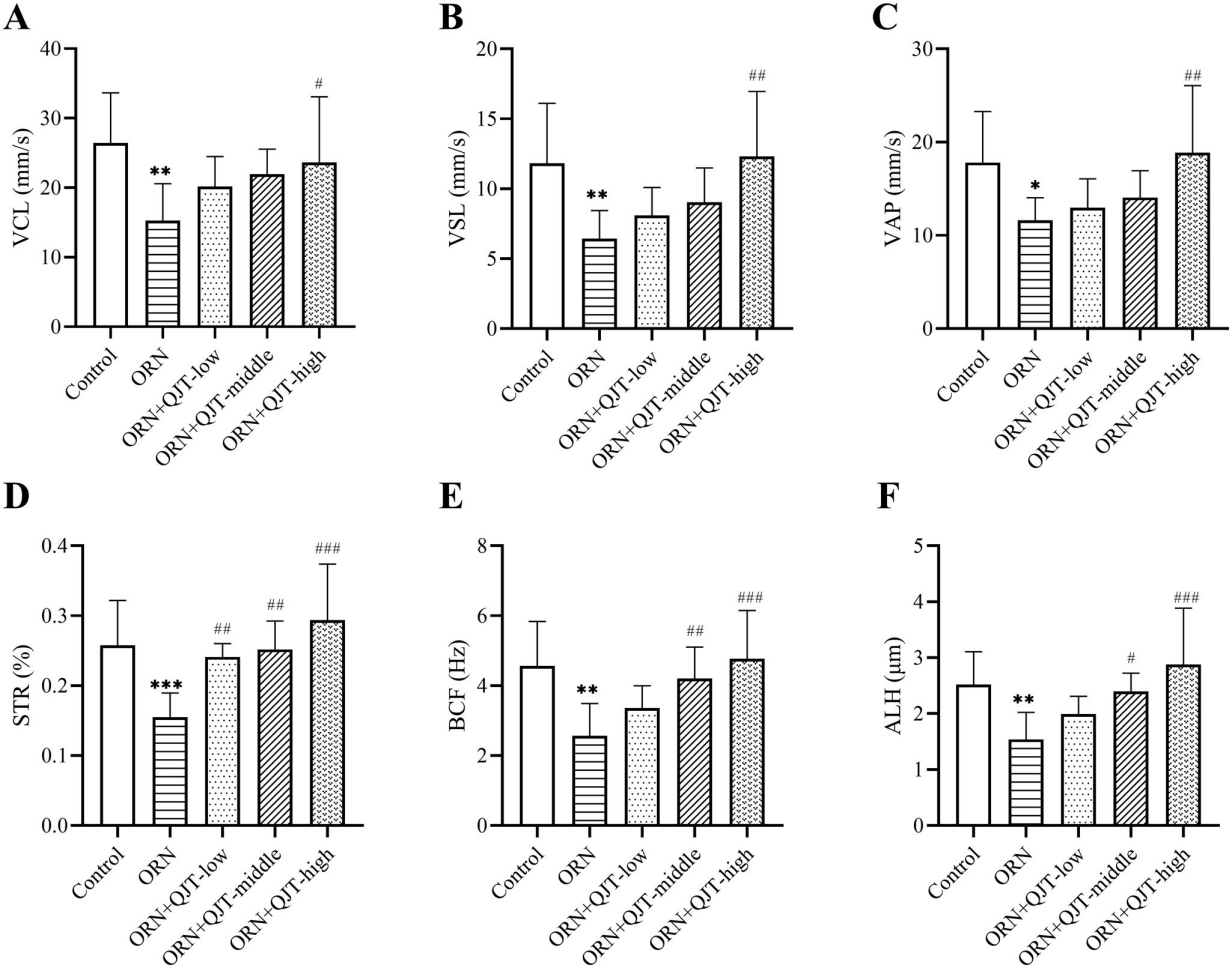
QJT treatment enhanced kinematic parameters of sperm. (A) Velocity curved line (VCL, mm/s), (B) velocity straight line (VSL, mm/s), (C) velocity average path (VAP, mm/s), (D) straightness (STR, %), (E) beat cross frequency (BCF, Hz), and (F) amplitude of lateral head displacement (ALH, μm). *p < 0.05 **p < 0.01, *vs.* Control group. ^#^*p* < 0.05 ^##^*p* < 0.01, ^###^*p* < 0.001, *vs.* ORN group.

As shown in Table 1, the sperm density, progressive and non-progressive motility (PR + NP) sperm, and PR grade sperm in rats treated with ORN were decreased compared with those in the control group, which were improved with the QJT treatment. High-dose administration of QJT showed the best effect on the amelioration of sperm parameters in ORN-treated rats. Moreover, only the high-dose QJT treatment increased the PR + NP ratio to over 40%. A consistent tendency was observed in the kinematic parameters of sperm (VCL, VSL, VAP, STR, BCF, and ALH) (Figure 1). Thus, the QJT treatment enhanced sperm motility, concentration, and viability in ORN-treated rats.

**Table 1.** The effects of QJT on semen density, motility and viability.

Group	Case numbers	Density (×10^6^/mL)	PR + NP (%)	PR(%)
Control	14	23.22 ± 5.52	44.5 ± 6.90	24.79 ± 8.83
ORN	14	19.77 ± 8.82	28.07 ± 9.34*	19.79 ± 6.93
ORN + QJT-low	15	19.74 ± 6.41	36.8 ± 3.08^#^	24.8 ± 6.55
ORN + QJT-middle	14	31.14 ± 21.85^#^	38.86 ± 3.66^#^	21.21 ± 7.1
ORN + QJT-high	15	30.31 ± 13.53^#^	46.93 ± 11.56^#^	25.4 ± 12.45

Values are expressed as mean ± SD, *n* = 15 per group. *p < 0.05 *vs.* Control group. ^#^*p* < 0.05 *vs.* ORN group.

### QJT diminished the pathology of testis and epididymis from ORN-treated rats

**Figure 2.**
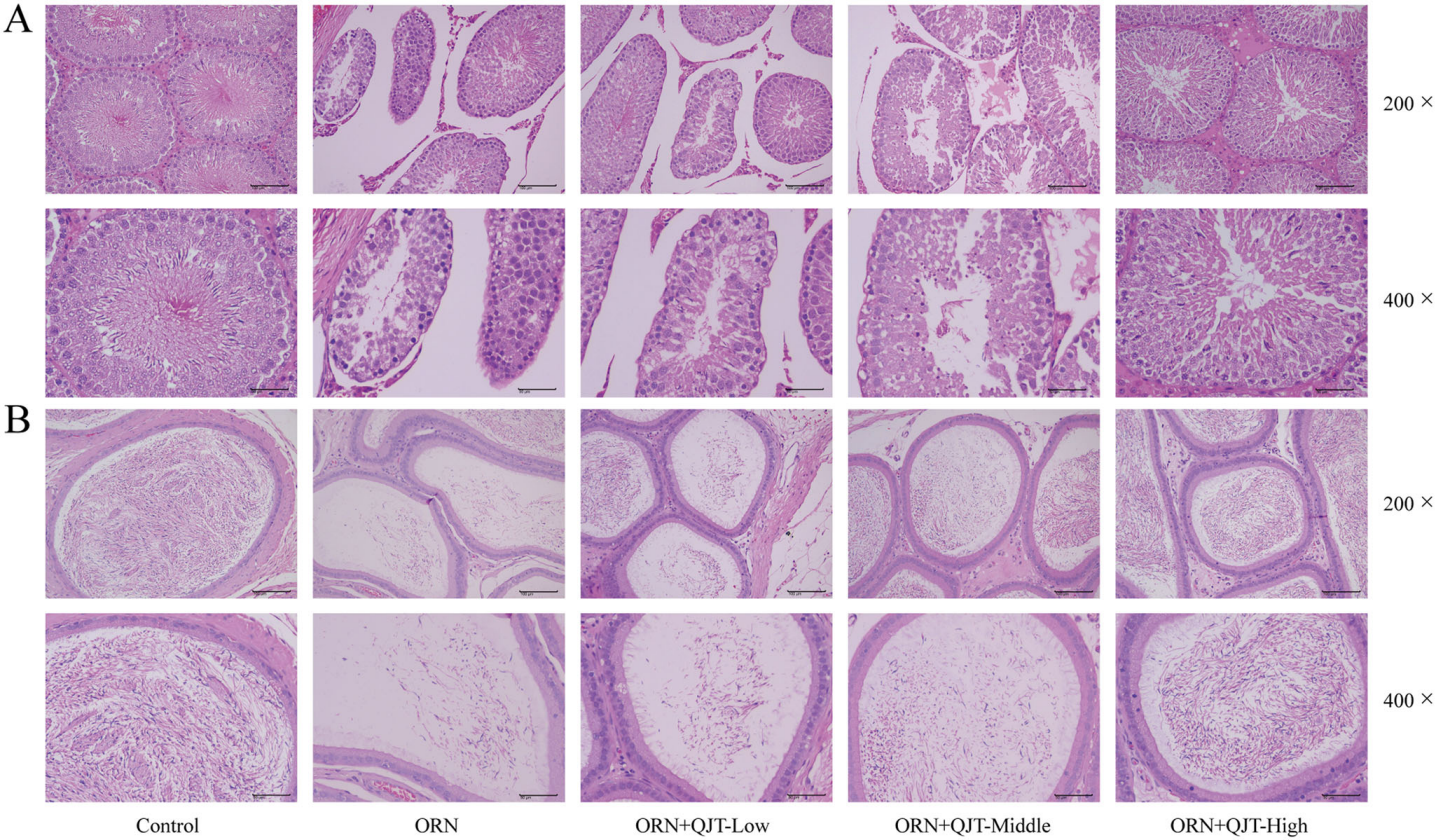
QJT alleviated the pathology of testis and epididymis in ORN-treated rats. Testis and epididymis tissues were assessed by H&E staining.

The effect of QJT on testicular function was assessed by the pathological staining. Based on the H&E results, in the model group, the parenchymal seminiferous tubules in the testicular tissue were different sizes; the seminiferous tubules in local areas were atrophic and necrotic with an obviously smaller volume and irregular shape; the spermatogenic cells were necrotic with a reduced number; the space of seminiferous tubules was larger, and interstitial cells were observed in the interstitium (Figure 2(A)). In the epididymal tissue, sperm level in the local lumen was significantly reduced and was almost absent in the severely affected area (Figure 2(B)). However, these pathological manifestations were mitigated by the QJT treatment, especially the high-dose QJT.

### QJT enhanced mitophagy and mitochondrial ubiquitination of ORN-treated rats

**Figure 3.**
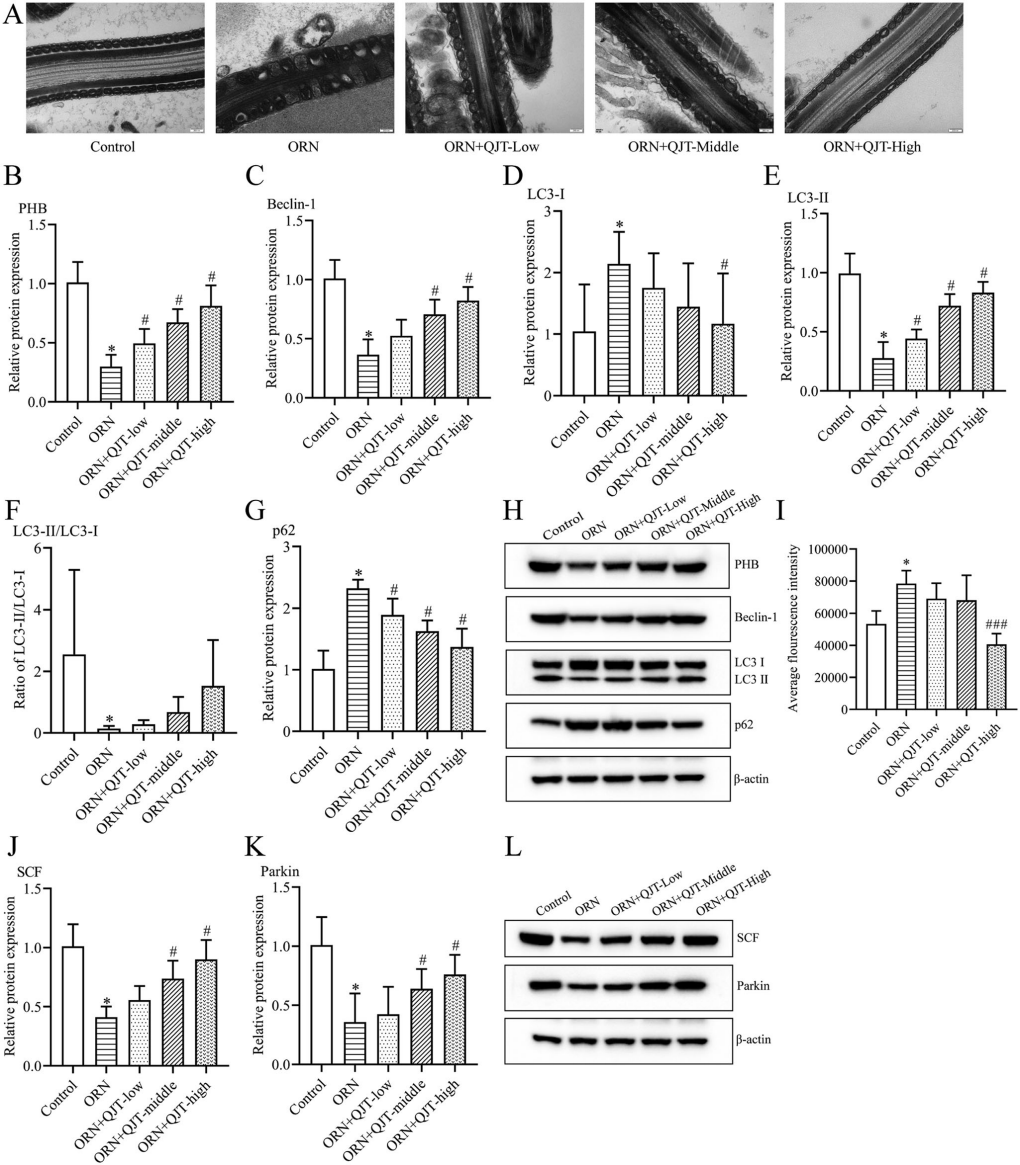
QJT increased mitophagy and mitochondrial ubiquitination of ORN-treated rats. (A) Mitochondria were evaluated by TEM. (B–H) Relative protein levels of PHB (B), Beclin-1 (C), LC3 (D–F) and p62 (G) were detected by western blotting. (I) ROS level was determined by a flow cytometer. (J–K) Relative protein levels of SCF (J) and Parkin (K) were measured by western blotting. Data were expressed after being normalized to β-actin. *p < 0.05 *vs.* Control group. ^#^*p* < 0.05 *vs.* ORN group. All assays were performed five times.

To explore the mechanisms of QJT on the ORN-treated AZS, we focused on mitophagy based on our previous findings. TEM revealed that most of the mitochondria in the tails of the sperm from ORN-treated rats were swollen; the cristae were dissolved, broken, or had disappeared; the morphological differences between the mitochondria were obvious; and some of the mitochondria were irregular in shape (Figure 3(A)). The QJT treatment consistently ameliorated this TEM results (Figure 3(A)). In addition, the relative protein expressions of PHB, Beclin-1 and LC3-II were significantly reduced, and the levels of p62 and LC3-I proteins were significantly increased in ORN-treated rats compared with those in control rats. The QJT treatment prominently reversed the ORN-treated changes in the levels of PHB, Beclin-1, and p62, and that of LC3-II/LC3-I was inverted with QJT treatment, with no statistical difference (Figure 3(B–H)). In addition, the QJT treatment enhanced the ORN-reduced ROS levels, although no statistical difference was also observed (Figure 3(I)). Moreover, the relative expressions of SCF and Parkin were also dramatically decreased in ORN-treated rats, which was significantly reversed by both the middle- and high-dose QJT treatment (Figure 3(J–K)). Therefore, these results indicate that the administration of QJT strengthened the mitophagy and mitochondrial ubiquitination in AZS rats.

### QJT regulated LKB1/AMPK/ULK1 signaling pathway in ORN-treated rats

**Figure 4.**
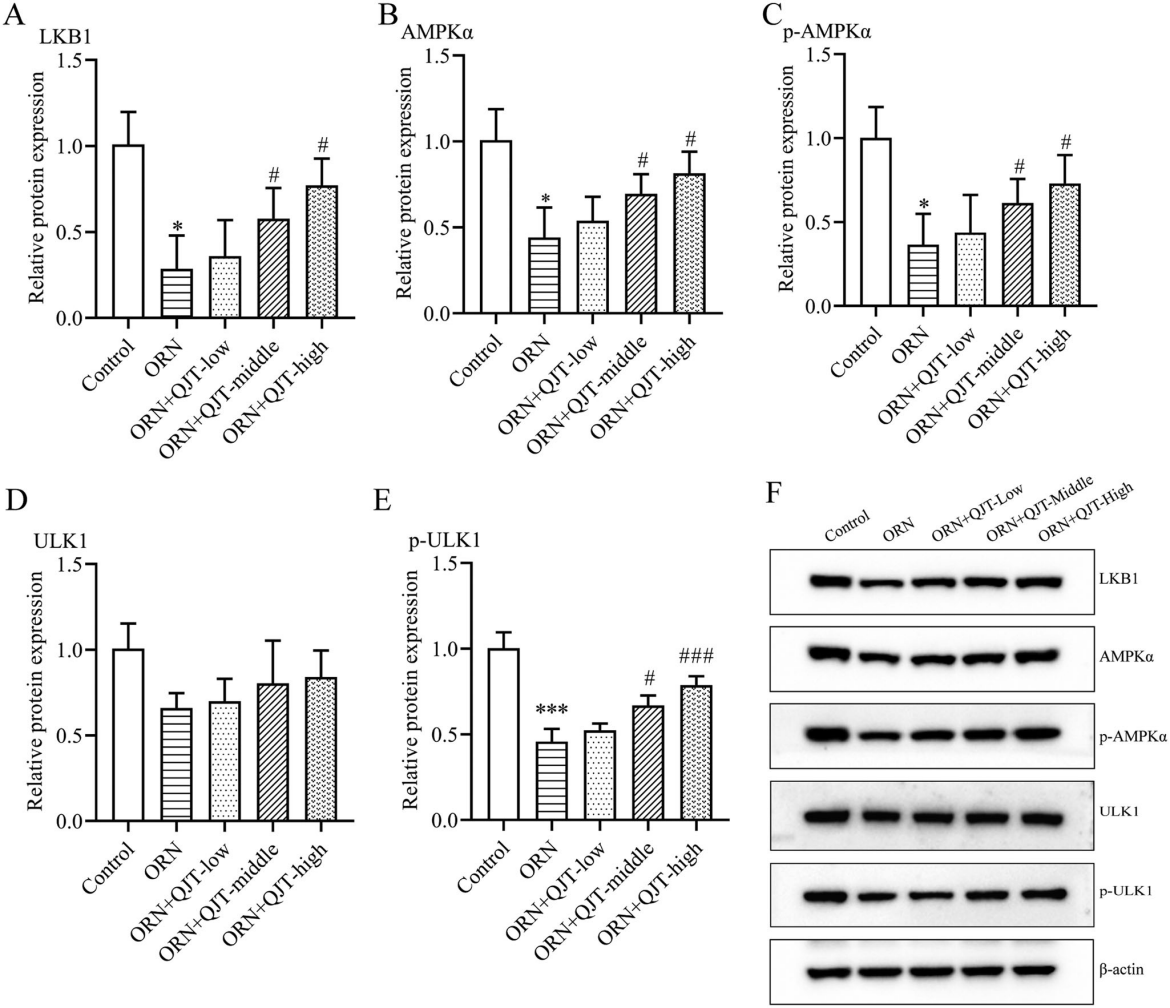
QJT regulated LKB1/AMPK signaling pathway in ORN-treated rats. (A–E) Relative protein levels of LKB1 (A), AMPKα (B), p-AMPKα (C), ULK1 (D), and p-ULK1 (E) were examined by western blotting. (F) Representative images of western blotting protein bands. Data were expressed after being normalized to β-actin. *p < 0.05 *vs.* Control group. ^#^*p* < 0.05 *vs.* ORN group. All assays were performed five times.

To further investigate the potential mechanism of QJT in ORN-treated AZS, we analyzed the expression level of the LKB1/AMPK/ULK1 signaling pathway. Western blotting results revealed that the relative protein expressions levels of LKB1, AMPKα, p-AMPKα and p-ULK1 were obviously downregulated in the ORN-treated rats, which was significantly reversed with the middle- and high-dose QJT treatment (Figure 4(A–F)). Although the relative protein expression levels of ULK1 were increased by QJT, there was no statistical difference. Hence, QJT modulated the LKB1/AMPK/ULK1 signaling pathway in ORN-treated rats.

### QJT promoted mitophagy and mitochondrial ubiquitination via the LKB1/AMPK/ULK1 signaling pathway in ORN-treated rats

**Figure 5.**
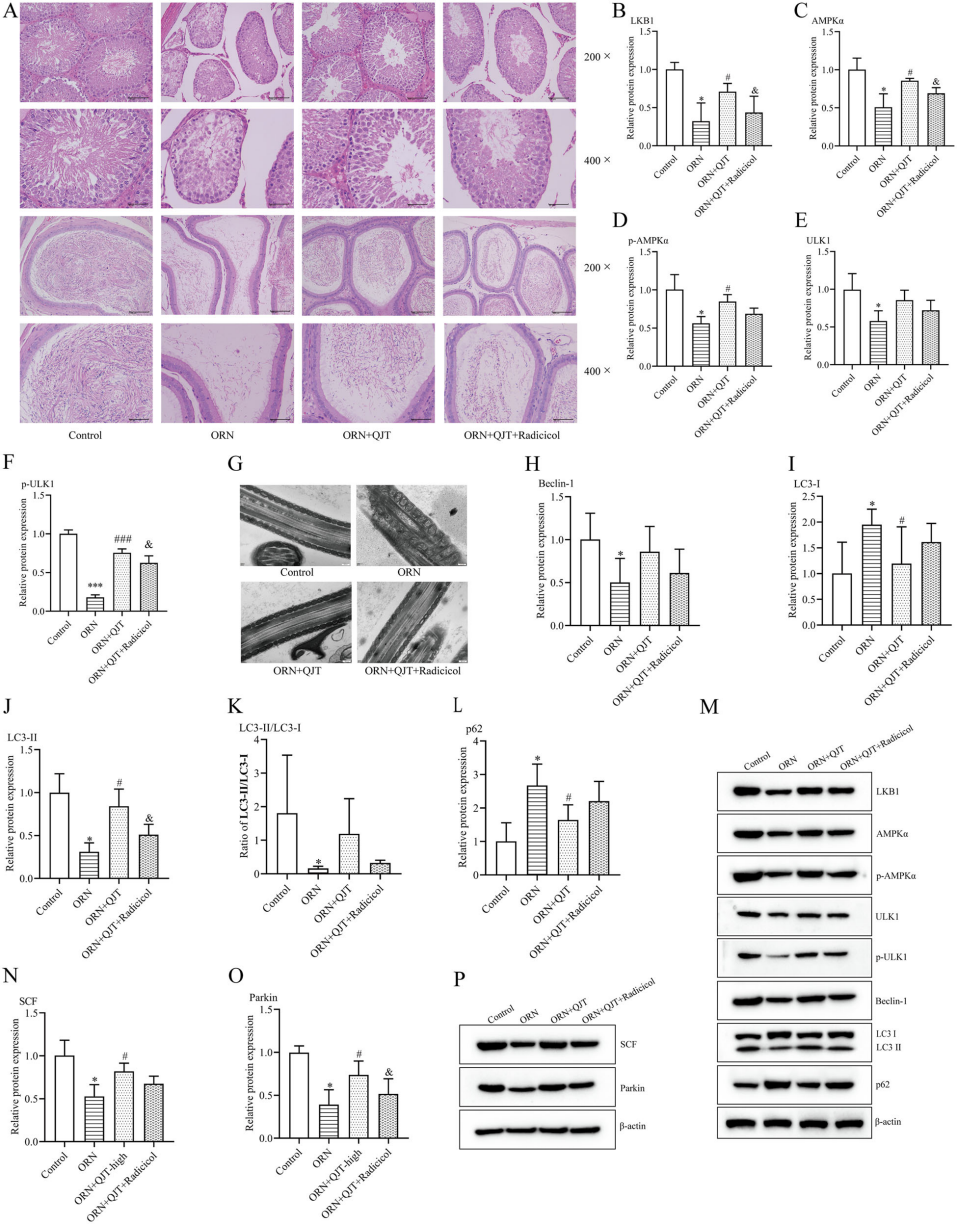
QJT increased mitophagy and mitochondrial ubiquitination by LKB1/AMPK signaling pathway in ORN-treated rats. (A) Testis (upper) and epididymis (down) tissues were assessed by H&E staining. (B–D) Relative protein levels of LKB1 (B), AMPKα (C), p-AMPKα (D), ULK1 (E), and p-ULK1 (F) were examined by western blotting. (G) Mitochondria were evaluated by TEM. (H–P) Relative protein levels of Beclin-1 (H), LC3 (I–K), p62 (L), SCF (N), and Parkin (O) were detected by western blotting. Data were expressed after being normalized to β-actin. *p < 0.05 vs. Control group; ^#^*p* < 0.05 *vs*. ORN group; ^&^*p* < 0.05 *vs*. ORN + QJT group. All assays were performed five times.

As indicated in Figure 5, the ameliorative effect of the QJT treatment on the pathological manifestations in ORN-treated rats was obviously neutralized by radicicol treatment (Figure 5(A)). Radicicol treatment was expected to significantly reduce the QJT-enhanced relative protein expressions levels of LKB1, AMPKα, and ULK1, while only decreasing the p-AMPKα and ULK1 levels, with no statistical difference in ORN-treated rats (Figure 5(B–F)). Furthermore, the improvement in mitochondrial morphology by QJT treatment was also significantly offset by radicicol treatment in ORN-treated rats (Figure 5(G)). The increased levels of Beclin-1, LC3-II, SCF, and Parkin, and the reduced expression of p62 caused by QJT treatment were also reversed by radicicol treatment in ORN-treated rats (Figure 5(H–P)). Thus, QJT enhanced mitophagy and mitochondrial ubiquitination *via* the LKB1/AMPK/ULK1 signaling pathway in ORN-treated rats.

## Discussion

AZS causes almost 20% of male infertility (Shay et al. 2016). The therapeutic effect of QJT, composed of several TCMs, on sperm vitality has been confirmed in the clinic (You et al. 2020). In our recent study, QJT improved AZS in male rats (Li G, Zhang, et al. 2021). However, the mechanism of action remains unclear. In this study, we found that QJT ameliorated AZS *via* mitochondrial ubiquitination and mitophagy mediated by the LKB1/AMPK/ULK1 signaling pathway.

Male fertility is highly dependent on sperm quality, with semen motility, concentration, vitality, and morphology being the dominant factors (Pan and Huang 2015). This study showed that QJT, especially high-dose QJT, significantly increased sperm motility, concentration, and viability in ORN-treated rats. This finding was consistent with our previous findings (Li G, Zhang, et al. 2021). Moreover, our H&E staining results revealed that QJT obviously alleviated ORN-treated pathological manifestations in testicular and epididymal tissues. These results indicated that QJT ameliorated sperm quality in ORN-treated rats.

Mitochondria are essential organelles that maintain energy, cell survival, and signaling, and are a main site of ROS production (Wang Y et al. 2012). The robustness of sperm is mainly sustained by ATP generated by mitochondrial aerobic oxidation (Eddy et al. 2003). When there is an excess of ROS, mostly produced by mitochondria, mitochondrial function becomes dysfunctional, destroying the structural integrity and quality of sperm (Sikka 2001). Our study showed that the ROS content in spermatozoa of ORN-treated AZS rats was significantly increased and decreased after the QJT treatment, indicating that QJT engaged in mitochondrial regulation and protected cells from excessive oxidative stress. Therefore, the reduced oxidative stress induced by QJT may be attributed to their protective effects on mitochondrial function. Previous reports have shown that mitochondrial dysfunction could result in lower sperm motility (Nowicka-Bauer et al. 2018). In this study, ORN induced impaired mitochondrial morphology, distinctly mitigated by the QJT treatment. PHB is an inner mitochondrial membrane phagocytic receptor that regulates mitochondrial function and maintains mitochondrial homeostasis (He L et al. 2017). LC3, P62, and Beclin-1 are autophagy-related proteins involved in the production of autophagosomes (Mizushima 2004). PHB interacts with the autophagosome-associated protein LC3 to induce autophagosome formation and autophagy development (Galluzzi et al. 2017). In this study, QJT increased the levels of PHB, Beclin-1, and LC3-II proteins, and reduced the relative protein expression level of p62, which suggested that the QJT treatment improved sperm quality by promoting mitophagy in ORN-treated rats. Moreover, studies have discussed mitophagy in post-fertilization sperm (Song et al. 2016). Eid et al. have documented that mitophagy was activated in ethanol-induced mitochondrial damage of Sertoli cells, which was associated with upregulation of Parkin related to male fertility (Eid and Kondo 2018; Eid et al. 2019). PINK1/Parkin-mediated signaling participates in the autophagy of damaged mitochondria, and substrate ubiquitination mediated by Parkin is a prerequisite process that induces mitophagy (Jin SM and Youle 2012; McWilliams and Muqit 2017). Protein ubiquitination during spermatogenesis was reviewed by Berruti (2021) recently. SCF ubiquitin ligases regulate various protein ubiquitination pathways and engage in mitochondrial damage and autophagy mechanisms (Yoshida et al. 2017). In addition, Parkin was found to be a component of an SCF-like ubiquitin ligase complex (Staropoli et al. 2003). In this study, the relative protein levels of SCF and Parkin were significantly downregulated in ORN-treated rats, which was antagonized by the QJT treatment, indicating that QJT also facilitated mitochondrial ubiquitination in ORN-treated rats. These findings clarify that QJT enhanced mitophagy and mitochondrial ubiquitination in ORN-treated rats.

Adenosine monophosphate protein kinase (AMPK) is a cellular energy sensor that participates in various pathological and physiological processes, including autophagy (Villanueva-Paz et al. 2016). In addition, AMPK participates in regulating mitochondrial homeostasis and oxidative stress (Thirupathi and de Souza 2017). When ROS is excessive, AMPK is activated through various mechanisms to inhibit oxidative stress-mediated mitochondrial ROS production and improves mitochondrial damage (Chen et al. 2018). Liver Kinase B1 (LKB1), upstream of AMPK, always acts as a switch to modulate AMPK activation (Jia et al. 2019). ULK1 is a substrate for AMPK phosphorylation, and the binding of AMPK to ULK1 plays a critical role in autophagy induction (Egan et al. 2011). The LKB1/AMPK/ULK1 signaling pathway has been implicated in mitochondrial function in various diseases and biological processes, such as sarcopenic obesity (Huang et al. 2019), primary rat hepatocytes exposure to the xenoestrogen bisphenol-A (Anand et al. 2020), colitis and colitis-associated cancer (Wang SQ, Cui, et al. 2019; Wang SQ, Yang, et al. 2019), cancer outcomes (Jiang et al. 2019; Jin C et al. 2021), and adipogenesis (He Y et al. 2013). Pang et al. (2018) showed that AMPK/ULK1 signal pathway mediated autophagy and apoptosis affect testis development. In addition, Duan et al. (2016) summarized the important role of the PI3K-Akt/LKB1-AMPK-mTOR signaling axis in testis development and spermatogenesis. In this study, the relative protein expression of LKB1, AMPKα, p-AMPKα, ULK1, and p-ULK1 was obviously down-regulated in the ORN-treated rats, which was significantly reversed by the QJT treatment. Moreover, the ameliorative effect of the QJT treatment on pathological manifestations, mitochondrial morphology, and the expressions of mitophagy and mitochondrial ubiquitination-related proteins was significantly counteracted by radicicol, an inhibitor of LKB1 (He Y et al. 2013). Therefore, the results suggest that ORN leads to mitochondrial damage by inducing excessive activation of ROS, and QJT activated the LKB1/AMPK/ULK1 signaling pathway, relieve oxidative stress, and promote mitophagy and ubiquitination to improve sperm motility in AZS rats.

An increasing number of studies have demonstrated the ameliorative effect of TCM on sperm quality (Wang SC et al. 2018). For instance, the BaZiBuShen formula improved spermatogenesis involved in the Sirt6/P53 and Sirt6/NF-κB signaling pathways (Li L, Chen, et al. 2021). In addition, taurine enhanced sperm quality and function in ORN-treated AZS rats (Du et al. 2019). QJT contains various TCMs with powerful chemical component that may be useful for treating AZS. The main active ingredient of *Ginseng* is mainly ginsenoside. Ginsenoside Rb1 regulates mitochondrial function by regulating ROS levels in mitochondria and preventing excessive production of ROS (Li J et al. 2012; Zhou et al. 2019). Studies have reported that *Lycium barbarum* polysaccharides significantly improve sperm viability, plasma membrane integrity and mitochondrial activity (Zhang R et al. 2022). *Angelica* polysaccharides reduce apoptosis and ROS, protect mitochondrial integrity, and improve energy metabolism by inhibiting the antioxidant effect of the caspase pathways (Zhuang et al. 2020). Icariin is the main active ingredient extracted from *Epimedium* and is commonly used to treat male dysfunction. It improves sperm defects in rats by increasing cell proliferation and inhibiting mitochondria-dependent apoptosis pathways, as well as exerting protective effects against testicular dysfunction (He W et al. 2021). *Psyllium* polysaccharide may be useful as a natural antioxidant (Yin et al. 2010). *Cuscuta sinensis* is a TCM with liver and kidney tonic properties that can improve kidney function (Shin et al. 2011). Leonurine, the active compound in *Leonurus japonicus*, can inhibit the production of mitochondrial ROS and restore mitochondrial function and redox status (Loh et al. 2010). *Curculigo orchioides* tonifies the kidney and helps Yang, it can improve sexual dysfunction induced by high sugar levels and increase semen parameters and sperm count in rats (Thakur et al. 2012). TCMs are frequently multi-component, multi-potent, multi-target, and multi-pathway modes of action. This study demonstrated that QJT treated AZS by improving mitochondrial function and antioxidant effects. However, the relationship between the components and the efficacy of QJT, as well as the mechanism, require further study.

## Conclusions

An AZS rat model was constructed by the induction of ORN, and the QJT treatment significantly increased sperm motility, concentration, and viability in ORN-treated rats, and alleviated the ORN-treated pathological manifestations in testicular and epididymal tissues. In addition, the QJT treatment distinctly mitigated the impaired mitochondrial morphology; increased the levels of PHB, Beclin-1, and LC3-II proteins, and reduced the relative protein expression of p62. Regarding the mechanism, the QJT treatment antagonized the down-regulated protein levels of SCF and Parkin. Moreover, QJT activated the LKB1/AMPK/ULK1 signaling pathway, relieved oxidative stress, and promoted mitophagy and ubiquitination to improve sperm motility in AZS rats. In brief, our findings provide a theoretical basis for the treatment of AZS and male fertility.
